# Transverse Analysis of Maxilla and Mandible in Adults with Normal Occlusion: A Cone Beam Computed Tomography Study

**DOI:** 10.3390/jimaging8040100

**Published:** 2022-04-05

**Authors:** Kyung Jin Lee, Hyeran Helen Jeon, Normand Boucher, Chun-Hsi Chung

**Affiliations:** Department of Orthodontics, School of Dental Medicine, University of Pennsylvania, 240 South 40th Street, Philadelphia, PA 19104-6030, USA; kl522@cornell.edu (K.J.L.); hjeon@upenn.edu (H.H.J.); nboucher@upenn.edu (N.B.)

**Keywords:** transverse width, inclination, first molar, CBCT, normal occlusion

## Abstract

Objectives: To study the transverse widths of maxilla and mandible and their relationship with the inclination of first molars. Materials and Methods: Fifty-six untreated adults (12 males, 44 females) with normal occlusion were included. On each Cone Beam Computed Tomography (CBCT) image of the subject, inter-buccal and inter-lingual bone widths were measured at the levels of hard palate, alveolar crest and furcation of the first molars, and maxillomandibular width differentials were calculated. In addition, the buccolingual inclination of each first molar was measured and its correlation with the maxillomandibular width differential was tested. Results: At the furcation level of the first molar, the maxillary inter-buccal bone width was more than the mandibular inter-buccal bone width by 1.1 ± 4.5 mm for males and 1.6 ± 2.9 mm for females; the mandibular inter-lingual bone width was more than the maxillary inter-lingual bone width by 1.3 ± 3.6 mm for males and 0.3 ± 3.2 mm for females. For females, there was a negative correlation between the maxillomandibular inter-lingual bone differential and maxillary first molar buccal inclination (*p* < 0.05), and a positive correlation between the maxillomandibular inter-lingual bone differential and mandibular first molar lingual inclination (*p* < 0.05). Conclusions: This is a randomized clinical study on transverse analysis of maxilla and mandible in adults with normal occlusion using CBCTs. On average: (1) At the furcation level of the first molars, the maxillary inter-buccal bone width was slightly wider than mandibular inter-buccal bone width; whereas the mandibular inter-lingual bone width was slightly wider than maxillary inter-lingual bone width; (2) A statistically significant correlation existed between the maxillomandibular transverse skeletal differentials and molar inclinations.

## 1. Introduction

In orthodontics, among the sagittal, vertical, and transverse planes of space, the transverse dimension is the least studied. There are many articles dealing with sagittal and vertical dimensions of the face in the orthodontic literature, but few related to the transverse dimension. Traditionally, transverse dimension has been studied using posteroanterior (PA) cephalograms and dental casts. In 1982, Ricketts et al. [1] used PA cephalogram to measure the transverse width of maxilla from the left jugale (J) to right jugale (J) and mandibular width from left antegonial notch (Ag) to right antegonial notch (Ag) and determined age-adjusted normative values. He reported that, at age 9, the maxillary width is 61.9 mm, with an increase of 0.6 mm per year, and mandibular width is 76.1 mm, with an increase of 1.4 mm per year, until age 16. In contrast, Ricketts and Grummons [2] reported an increase in J-J distance from 55 to 73 mm or 1 mm per year, and an increase in Ag-Ag distance from 68 to 94 mm or 1.5 mm per year in males, from age 3 to 21. In a later study using PA and lateral cephalograms, Wagner and Chung [3] found that there is a relationship between the transverse growth and vertical facial types. At age 6, the dolichofacial subjects had smaller maxillary (J-J) widths and mandibular (Ag-Ag) than brachyfacial subjects and this trend continued until age 18. Recent studies introduced the concept of the Total Face Approach (TFA) 3D cephalometric analysis system using the cone beam computed tomography (CBCT) [4,5,6]. The 3D cephalometric analysis provided reliable information in locating skeletal discrepancies and determining skeletal intermaxillary relation via plane and specific point references with the limitation of a small sample size [6].

Using dental casts, McNamara et al. [7] studied the maxillary transpalatal width, the distance from the gingival margin of lingual groove of the first molar to the contralateral side, reporting mean values of 32.7 mm at age 7, 33.2 mm at age 8, 33.2 mm at age 9, 33.7 mm ate age 10, 34.5 mm at age 11, and 35.2 mm at age 12. After age 12, there was no change in the transpalatal width. Andrews [8] analyzed dental casts and suggested that the WALA ridge, which is coincident with the mucogingival junction, determined the width of the mandible. He stated that optimally positioned mandibular molars should be upright in the alveolus, and their FA point (center of the crown) should be horizontally positioned 2 mm from the WALA ridge.

However, there are limitations when using PA cephalograms and dental casts. For example, the superimposition of many structures on the posteroanterior view reduces the clarity of the landmarks and increases identification errors [9,10]. Any rotation of tipping of the head when taking the PA cephalogram affects the horizontal relationships of the landmarks, making it hard to assess symmetry and measure horizontal distances [11]. The landmarks located farther from the posteroanterior porionic axis also have greater variations and are less reliable in evaluating the transverse dimension than closer landmarks [12]. As for the dental casts, they have the limitations to precisely reveal the skeletal dimensions of maxilla and mandible, the basal bone and root positions in the alveolar bone. 

The advent of the CBCT produces minimum distortion, no magnification, and eliminates all the problems of superimposition. Thus, the widths of maxillary and mandibular bones and their relationship to each other, the buccolingual inclination of each posterior tooth, and their root positions in the alveolar bone, can be clearly visualized. In addition, a recent study supports that CBCT is useful in assessing soft tissue facial characteristics [13].

Some studies have used CBCT to analyze the molar inclinations and transverse widths of maxilla and mandible. Alkhatib and Chung [14] found the maxillary first molar had a mean buccal inclination of 4.9° and the mandibular first molar had a mean lingual inclination of 12.6° in adults with normal occlusion. Barrera et al. [15] reported in their 10 adult subjects that the maxillary second molars had a buccal tipping of 12.5°. Yang and Chung [16] measured the buccolingual inclination of maxillary and mandibular first molars in patients aged 6–9 years, 9–10 years, and 23–25 years. They concluded that maxillary first molars exhibited buccal inclination in all groups, and the adults displayed less inclination than children; the mandibular first molars exhibited lingual inclination, and the adults displayed less inclination than did children. Yi et al. [17] evaluated the longitudinal transverse growth of the maxilla-mandibular complex in untreated children using CBCT. They concluded that the maxillary and mandibular molars upright with age, and the maxillary and mandibular intermolar widths, when measured from the buccal cusp tips, increase at an equal rate.

More recently, Miner et al. [18,19] analyzed CBCT images of subjects with and without crossbite to assess the width of the jaws and the inclination of the first molars. They reported that in normal occlusion, at the mid-alveolar bone levels of lingual surfaces of the first maxillary and mandibular first molars, the maxillary width is 1.2 ± 2.9 mm less than mandibular width with a wide range.

The purpose of this study is to investigate the transverse skeletal widths of maxilla and mandible at different levels of the first molar, and their relationship with molar inclinations in adults with normal occlusion using cone beam computed tomography (CBCT).

## 2. Materials and Methods

Institutional review board approval (#830003) was obtained from the University of Pennsylvania prior to collecting any CBCT information. The CBCT images were previously taken at a private practice with an I-CAT machine (Imaging Sciences International, Hatfield, PA, USA) in 0.3 mm voxel size. All CBCT images were oriented and analyzed using the Dolphin Imaging 3D software (version 10.5, Dolphin Imaging and Management Solutions, Chatsworth, CA, USA).

### 2.1. Sample

Fifty-six untreated adult subjects (12 males, 44 females) were selected (mean age: 35.4, range: 18 to 65). The inclusion criteria were: (1) no prior orthodontic treatment; (2) less than 5mm of crowding or spacing per arch; (3) maxillary and mandibular first molars fully erupted and roots completely formed; (4) minimal dental wear; (5) skeletal Class I (ANB 0–4°); (6) no missing teeth other than third molars. The exclusion criteria were: (1) posterior crossbite; (2) posterior or anterior open bite; (3) crowns or significant restorations on any first molars; (4) presence of primary or supernumerary teeth; and (5) craniofacial deformities.

### 2.2. Orientation

Using Dolphin Imaging (version 10.5, Dolphin Imaging and Management Solutions, Chatsworth, CA, USA), each image was standardized and oriented such that the functional occlusal plane and a line connecting the inferior border of the orbital rims was parallel to the floor. The sagittal guideline of the tooth axis was defined as a line passing through the midpoint of the mesiodistal crown width and the midpoint of the mesiodistal width of the crown at the level of the furcation, as reported by Matsumoto et al. [20]. Once the sagittal orientation was determined, the coronal cross-section was obtained in a 0.5-mm slice, using a section that best fit the right and left molar mesiodistal midpoints. The coronal section was used to measure the bone widths and tooth inclinations.

### 2.3. Transverse Width Measurements

**Figure 1:**
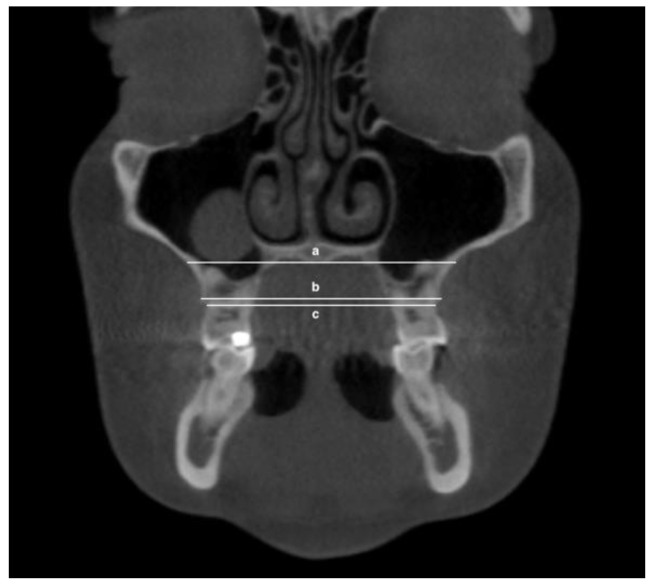
Maxillary inter-buccal bone widths at the level of hard palate (a), furcation (b) and alveolar crest (c).

**Figure 2:**
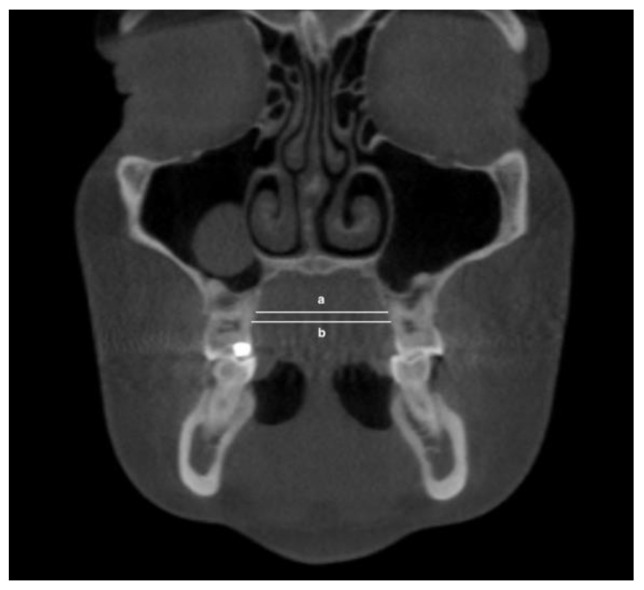
Maxillary inter-lingual bone widths at the level of furcation (a) and alveolar crest (b).

**Figure 3:**
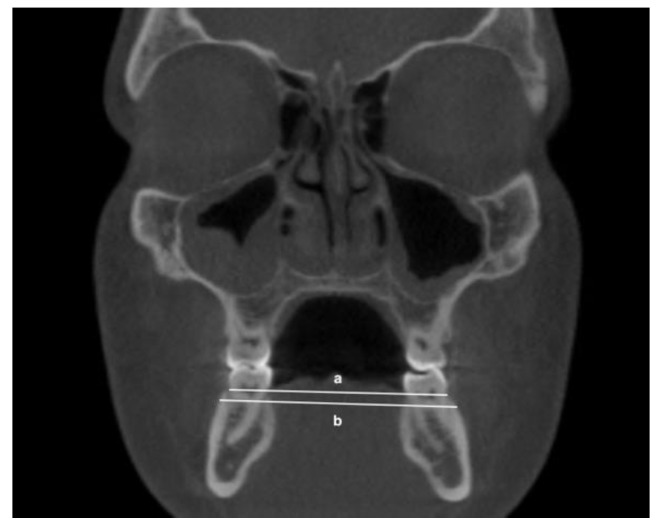
Mandibular inter-buccal bone widths at the level of alveolar crest (a) and furcation (b).

**Figure 4:**
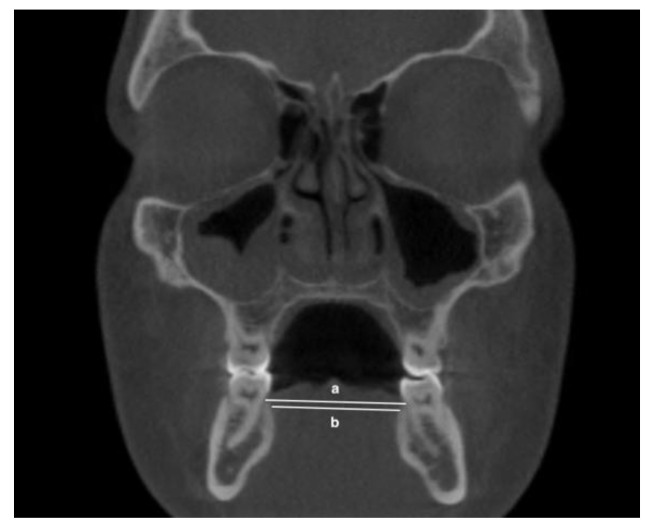
Mandibular inter-lingual bond widths at the level of alveolar crest (a) and furcation (b).

The following transverse width measurements were made as shown in Figure 1, Figure 2, Figure 3 and Figure 4:

- Maxillary inter-buccal bone widths: from the right to the left points at the level of hard palate, alveolar crest and furcation (Figure 1)
- Maxillary inter-lingual bone widths: from the right to the left points at the level of alveolar crest and furcation (Figure 2)
- Mandibular inter-buccal bone widths: from the right to the left points at the level of alveolar crest and furcation (Figure 3)
- Mandibular inter-lingual bone widths: from the right to the left points at the level of alveolar crest and furcation (Figure 4)
- The differentials of maxillomandibular inter-buccal bone or inter-lingual bone widths were calculated by subtracting mandibular measurements from maxillary measurements at the same level of the first molar.

### 2.4. Tooth Inclination Measurements

**Figure 5:**
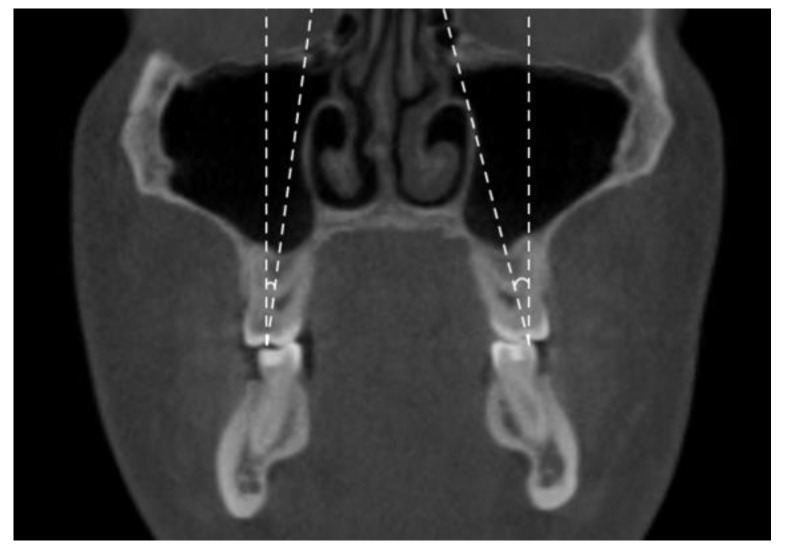
Inclination of maxillary first molars.

**Figure 6:**
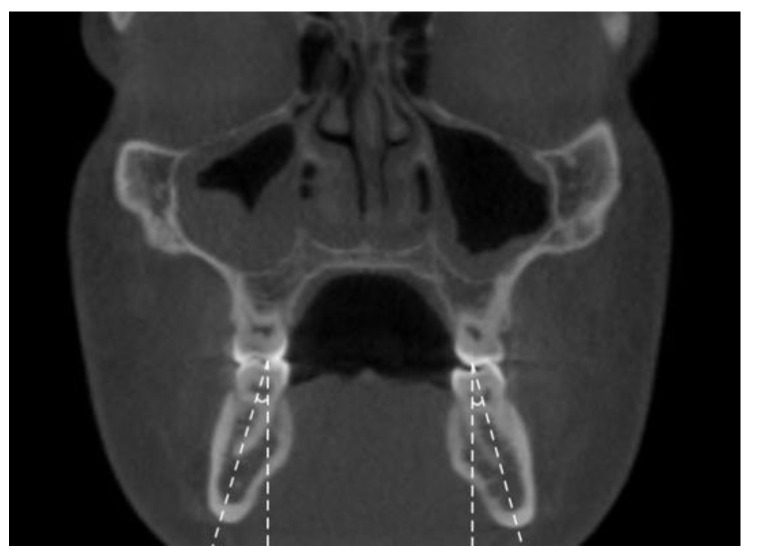
Inclination of mandibular first molars.

The following first molar inclination measurements were made as shown in Figure 5 and Figure 6. The long axis of the tooth was defined as a line connecting the midpoint of the buccal and lingual cusp tips and the midpoint of the buccolingual width at the cervical base close to the furcation of the anatomic crown. The angle was measured from the long axis of each maxillary and mandibular first molar to a vertical reference line that was perpendicular to the horizontal reference line. If the crown was lingual to the roots, the inclination would be negative (−) and if the crown was buccal to the roots, the inclination would be positive (+).

### 2.5. Statistical Analysis

Ten randomly selected subjects were measured again by the same examiner (K.J.L.) 3 weeks after the test, for intraexaminer reproducibility. A paired *t*-test was run for transverse skeletal and tooth inclination measurements to determine whether there were significant differences between the original measurements and the second measurements. The mean, standard deviation and range were calculated for each bone width and inclination. A regression analysis was tested between the maxillomandibular skeletal width differentials and molar inclinations. The significance was determined at *p* < 0.05.

## 3. Results

The intraexaminer reliability test showed no significant differences between original and repeated bone width and tooth inclination measurements (*p* > 0.05). The Pearson correlation coefficient varied between 0.90 and 0.99 for the measurements, indicating high reproducibility.

### 3.1. Transverse Width Measurements

The maxillary and mandibular inter-buccal bone and inter-lingual bone widths at the levels of hard palate, alveolar crest, and furcation of the male group are shown in Table 1, and the female group in Table 2. All of the measurements of the male group are greater than the female group.

### 3.2. Differentials of Transverse Width Measurements

The differentials of maxillomandibular inter-buccal bone and inter-lingual bone widths at the levels of alveolar crest and furcation are shown in Table 3. For males, the differentials of inter-buccal bone widths displayed an average of 0.9 mm ± 3.2 mm at the level of alveolar crest and 1.1 mm ± 4.5 mm at the level of furcation; the differentials of inter-lingual bone widths displayed an average of −0.4 mm ± 4.1 mm at the level of alveolar crest and −1.3 mm ± 3.6 mm at the level of furcation. For females, the differentials of inter-buccal bone widths displayed an average of 1.1 mm ± 2.2 mm at the level of alveolar crest and 1.6 mm ± 2.9 mm at the level of furcation; the differentials of inter-lingual bone widths differentials displayed an average of −0.6 mm ± 2.4 mm and −0.3 mm ± 3.2 mm at the level of furcation.

### 3.3. Tooth Inclination Measurements

Table 4 shows that the maxillary right first molar displayed a buccal inclination of 4.2° ± 5.9° and the maxillary left first molar displayed a buccal inclination of 6.5° ± 6.3°, and the average was 5.3° ± 6.2°. There was no statistically significant difference between the maxillary right and left measurements (*p >* 0.05). The mandibular right first molar displayed a lingual inclination of −13.9° ± 5.1° and the mandibular left first molar displayed a lingual inclination of −14.9°± 5.2°, and the average was −14.4° ± 5.2°. There was no statistically significant difference between the mandibular right and left measurements (*p >* 0.05).

### 3.4. Correlation between Differentials of Transverse Width and Tooth Inclination

The correlation between maxillomandibular differential values and maxillary and mandibular first molar inclination was tested. For males, the inter-buccal bone width differentials at the level of alveolar crest and furcation are positively correlated with mandibular first molar inclination, and the inter-lingual bone width differentials at the level of alveolar crest and furcation are positively correlated with mandibular first molar inclinations (*p* < 0.05). For females, the inter-buccal bone differentials at the level of alveolar crest and inter-lingual bone differentials at the level of alveolar crest and furcation are negatively correlated with maxillary upper molar inclinations (*p* < 0.05); the inter-lingual bone differentials at the level of alveolar crest and furcation are positively correlated with mandibular first molar inclinations (*p* < 0.05).

## 4. Discussion

In our study, there were 44 female subjects, but only 12 male subjects were included. The small sample size of the male group was due to limited subjects with normal occlusion that fit our inclusion criteria. A bigger sample size in the male group is needed in future studies.

One of the advantages of using CBCT to determine the buccolingual inclination of molars is the ability to visualize the whole tooth and avoid biases associated with tooth morphology or uneven wear of the cusps. Determining the tooth axis for the upper molars poses challenges due to the frequent divergence between the upper molar roots. There were no statistically significant differences noted between the male and female groups on the measurements of the buccolingual inclination of first molars. Thus, the male and female data were combined in our study.

We found that the measurements at the level of furcation are more reliable than at the alveolar crest due to the burnout of bone crest on the images. We did our best to find the first visible points at the alveolar crest level. The lingual measurements are also more reliable than the buccal measurements. The reason is that some subjects exhibited the presence of significant buccal shelf on the molar regions of the mandible, which made it very difficult to do the accurate measurements. In those cases, we measured the minimum distance at the intersecting points between the horizontal line passing through furcation points and mandibular buccal bone margin. Thus, it is suggested that the average lingual width measurements at furcation could be used as a standard when making a transverse diagnosis.

Interestingly, we found that at the furcation level of the first molar, the inter-lingual bone widths of maxilla and mandible are similar. On average, for the female group, almost all of the widths were the same (−0.3 ± 3.2 mm), and for the male group, the maxilla was 1.3 ± 3.6 mm narrower than mandible. Our data support the findings of Miner et al. [18], who reported that in normal occlusion, at the mid-alveolar bone levels of lingual surfaces of the first maxillary and mandibular first molars, the maxillary width is about 1.2 ± 2.9 mm less than mandibular width. It should be noted that the standard deviation is quite large, which can be attributed to the irregular shape of the maxillary and mandibular alveolar bones. 

In our study, we found that for both males and females, maxillary first molars had a buccal inclination of 5.3 ± 6.2°, and mandibular first molars had a lingual inclination of 14.4 ± 5.2°. Our data support the findings of Alkhatib and Chung [14], who reported that in male and female adults with normal occlusion, the maxillary molars had a buccal inclination (4.9° each) and mandibular molars had a lingual inclination (12.6° each). Our data also support the findings of Yang and Chung [16], who reported that for adults, the maxillary first molar exhibited about 4.7° buccal inclination and mandibular first molars exhibited about 13° lingual inclination. It has been anecdotally stated that the buccal inclination of maxillary molars and lingual inclination of mandibula molars (curve of Wilson) contributes to the most effective use of cuspal contacts, while avoiding nonfunctional contacts known as balancing interferences [21,22]. When the curve of Wilson is made too flat, ease of masticatory function may be impaired [23].

The relationship between the buccolingual inclination of molars and vertical facial type has been reported, with inconsistent findings. For example, Janson et al. [24] found that there was no statistically significant relationship between mandibular posterior teeth inclination and facial type but that maxillary molars had greater buccal inclination in high angle subjects. In contrast, Tsunori et al. [25] reported that short facial type individuals had more lingually inclined mandibular molars.

Our data showed that there was a correlation between the maxillomandibular bone width differential and buccolingual inclination of first molars. When the maxillary skeletal width is significantly narrower than the mandibular width, the maxillary molars tend to incline more buccally and the mandibular molars tend to incline more lingually to compensate. On the contrary, when the maxillary skeletal width is significantly wider than the mandibular width, the maxillary molars tend to incline more lingually and the mandibular molars tend to incline more buccally to compensate. This underlines the importance of evaluating the buccolingual inclinations of molars when making the transverse diagnosis on a patient clinically.

In our study, it is suggested that the use of CBCT is an appropriate process for establishing a transverse diagnosis to ensure proper skeletal widths of the maxilla and mandible in order to provide more accurate treatment planning and more favorable outcomes. As a result of these treatment objectives, the roots of teeth are positioned in the center of alveolar bones with buccal and lingual bone coverage and the teeth have proper inclinations to accommodate the shape of the bone. 

Our data suggested normal values in the diagnosis of transverse dimension when the CBCTs are available. In addition, we only provided data for adults and more studies are needed for the norms of children at different ages. 

## 5. Conclusions

In adults with normal occlusion, the following conclusions can be drawn from this study:

- Males’ maxillary and mandibular inter-buccal bone and inter-lingual bone widths on the first molars are wider than the corresponding females’ widths.
- At the level of furcation of the first molars, on average, maxillary inter-buccal bone width is slightly wider than mandibular buccal width by 1.1 mm for males and 1.6 mm for females. The maxillary inter-lingual bone width is slightly narrower than mandibular lingual width, by 1.3 mm for males and 0.3 mm for females.
- Maxillary first molars exhibited buccal inclination of 5.3°, whereas the mandibular first molar exhibited lingual inclination of 14.4°.
- A correlation exists between the maxillomandibular differential of inter-buccal and inter-lingual widths and upper and lower first molar inclinations.

## Figures and Tables

**Table 1:** Measurements of male inter-buccal and inter-lingual bone widths of maxilla and mandible.

Measurement	n	Mean (mm)	SD ^a^ (mm)	Min (mm)	Max (mm)
Maxillary inter-buccal bone width at hard palate	12	62.4	3.9	57.0	68.1
Maxillary inter-buccal bone width at alveolar crest of first molar	12	54.7	3.5	47.5	62
Maxillary inter-buccal bone width at furcation of first molar	12	58.9	3.5	52.2	66.1
Maxillary inter-lingual bone width at alveolar crest	12	34.8	3.4	27.6	41.4
Maxillary inter-lingual bone width at furcation of first molar	12	30.9	2.8	24.5	33.7
Mandibular inter-buccal bone width at alveolar crest of first molar	12	53.8	2.7	50.0	58.4
Mandibular inter-buccal bone width at furcation of first molar	12	57.8	3.0	53.3	62.8
Mandibular inter-lingual bone width at alveolar crest of first molar	12	35.1	2.6	30.1	40.3
Mandibular inter-lingual bone width at furcation of first molar	12	32.2	2.9	27.7	37.5

^a^ SD indicates standard deviation.

**Table 2:** Measurements of female inter-buccal and inter-lingual bone widths of maxilla and mandible.

Measurement	n	Mean (mm)	SD ^a^ (mm)	Min (mm)	Max (mm)
Maxillary inter-buccal bone width at hard palate	44	58.7	3.7	51.7	68.3
Maxillary inter-buccal bone width at alveolar crest of first molar	44	52.7	2.8	44.9	60.4
Maxillary inter-buccal bone width at furcation of first molar	44	56.5	3.5	49.1	66.8
Maxillary inter-lingual bone width at alveolar crest of first molar	44	32.9	2.4	26	40.1
Maxillary inter-lingual bone width at furcation of first molar	44	29.5	2.3	24.1	34.2
Mandibular inter-buccal bone width at alveolar crest of first molar	44	51.6	2.9	43.3	58.0
Mandibular inter-buccal bone width at furcation of first molar	44	54.9	3.2	47.2	61.1
Mandibular inter-lingual bone width at alveolar crest of first molar	44	33.5	2.4	28.0	38.1
Mandibular inter-lingual bone width at furcation of first molar	44	29.8	2.9	22.7	36.3

^a^ SD indicates standard deviation.

**Table 3:** Male and female differentials of maxillomandibular intra-buccal and intra-lingual bone widths at alveolar crest and furcation of the first molars.

**Differentials for Male**	**Mean (mm)**	**SD ^a^ (mm)**	**Min (mm)**	**Max (mm)**
Intrer-buccal bone width at alveolar crest	0.9	3.2	−4.7	6.0
Inter-buccal bone width at furcation	1.1	4.5	−7.3	7.0
Inter-lingual bone width at alveolar crest	−0.4	4.1	−9.4	5.7
Inter-lingual bone width at furcation	−1.3	3.6	−10	3.1
**Differentials for Female**	**Mean (mm)**	**SD ^a^ (mm)**	**Min (mm)**	**Max (mm)**
Inter-buccal bone width at alveolar crest	1.1	2.2	−3.3	7.8
Inter-buccal bone width at furcation	1.6	2.9	−4.6	8.4
Inter-lingual bone width at alveolar crest	−0.6	2.4	−6.7	4.4
Inter-lingual bone width at furcation	−0.3	3.2	−5.9	7.8

^a^ SD indicates standard deviation.

**Table 4:** Means, standard deviations, and ranges for the buccolingual inclinations of maxillary and mandibular. First molars (females and males combined).

Measurement	n	Mean (°)	SD ^a^ (°)	Min (°)	Max (°)
Maxillary right first molar	56	4.2	5.9	−10.5	16.1
Maxillary left first molar	56	6.5	6.3	−9.2	21.6
Average, maxillary first molar	112	5.3	6.2	−10.5	21.6
Mandibular right first molar	56	−13.9	5.1	−23.4	−5.5
Mandibular left first molar	56	−14.9	5.2	−31.4	−2.5
Average, mandibular first molar	112	−14.4	5.2	−31.4	−2.5

^a^ SD indicates standard deviation; (−) value indicates lingual inclination; (+) indicates buccal inclination.

## Data Availability

The data presented in this study are available on request.
